# Beyond Protection: The Cytotoxic Effect of Anti-Tat Antibodies in People Living with HIV

**DOI:** 10.3390/ijms26157229

**Published:** 2025-07-26

**Authors:** Juan Ernesto Gutiérrez-Sevilla, Jorge Gaona-Bernal, Gracia Viviana González-Enríquez, Martha Escoto-Delgadillo, Guillermo Moisés Zúñiga-González, Belinda Claudia Gómez-Meda, Silvia Gabriela Luévano-Gómez, Alma Minerva Pérez-Ríos, Maribel Ávila-Morán, Víctor Eduardo García-Arias, Jessica Paloma Torres-Ríos, Jhonathan Cárdenas-Bedoya, Blanca Miriam Torres-Mendoza

**Affiliations:** 1 Laboratorio de Inmunodeficiencias y Retrovirus Humanos, Centro de Investigación Biomédica de Occidente, Instituto Mexicano del Seguro Social, Guadalajara 44340, Mexico; juan251995@hotmail.com (J.E.G.-S.); martha.escotod@gmail.com (M.E.-D.); pal.tor90@gmail.com (J.P.T.-R.); jhonbedoya148@outlook.com (J.C.-B.); 2 Doctorado en Microbiología Médica, Centro Universitario de Ciencias de la Salud, Universidad de Guadalajara, Guadalajara 44340, Mexico; 3 Departamento de Microbiología y Patología, Centro Universitario de Ciencias de la Salud, Universidad de Guadalajara, Guadalajara 44340, Mexico; jorge.gaona@academicos.udg.mx; 4 Departamento de Disciplinas Filosóficas Metodológicas e Instrumentales, Centro Universitario de Ciencias de la Salud, Universidad de Guadalajara, Guadalajara 44340, Mexico; gracia.gonzalez@academicos.udg.mx; 5 Centro Universitario de Ciencias Agropecurarias, Universidad de Guadalajara, Guadalajara 45200, Mexico; 6 Laboratorio de Mutagénesis, Centro de Investigación Biomédica de Occidente, Instituto Mexicano del Seguro Social, Guadalajara 44340, Mexico; mutagenesis95@hotmail.com (G.M.Z.-G.); victorega85@gmail.com (V.E.G.-A.); 7 Departamento de Biología Molecular y Genómica, Instituto de Genética Humana “Dr. Enrique Corona Rivera”, Centro Universitario de Ciencias de la Salud, Universidad de Guadalajara, Guadalajara 44340, Mexico; belinda.gomez@academicos.udg.mx; 8 UMAE Hospital de Especialidades, Centro Médico Nacional de Occidente, Instituto Mexicano del Seguro Social, Guadalajara 44340, Mexico; silvia.luevanog@imss.gob.mx (S.G.L.-G.); maribel.avilam@imss.gob.mx (M.Á.-M.); 9 Servicio de Infectología, Hospital General Regional 110, Instituto Mexicano del Seguro Social, Guadalajara 44100, Mexico; a.perezrio@imss.gob.mx

**Keywords:** HIV, cytotoxicity, DNA damage, oxidative stress, antibodies, Tat protein

## Abstract

Although ART leads to viral suppression, people living with HIV (PLWH) still face an increased risk of comorbidities, such as cancer. The HIV-1 Tat protein may contribute to the promotion of chronic inflammation, oxidative stress, and genomic instability. While the presence of anti-Tat antibodies has been associated with slower disease progression, their potential role in modulating DNA damage remains unclear. Assess the effect of anti-Tat antibodies on cytotoxic and DNA damage in PLWH. A cross-sectional study was conducted in 178 PLWH. Serum anti-Tat IgG antibodies were measured using enzyme-linked immunosorbent assay (ELISA). Cytotoxicity and DNA damage were assessed via serum 8-hydroxy-2′-deoxyguanosine (8-OHdG) and nuclear anomalies (Micronucleus cytome assay) in 2000 buccal cells. Statistical significance was considered at *p* < 0.05. Anti-Tat antibodies were found in 24.2% of participants. Positive individuals had lower CD4+ T cell counts (*p* = 0.045) and higher levels of pyknosis (*p* = 0.0001). No differences in 8-OHdG were found, but 8-OHdG correlated positively with CD4+ counts (rho = 0.334, *p* = 0.006). Pyknosis negatively correlated with CD4+ counts (rho = −0.272, *p* = 0.027). Anti-Tat antibodies may not prevent DNA damage but could be related to cytotoxic effects in PLWH.

## 1. Introduction

Despite the significant improvements in prognosis and quality of life for people living with HIV (PLWH) brought about by antiretroviral therapy (ART), which suppresses viral replication and promotes immune recovery, several immunopathological processes persist. Numerous studies have reported sustained immune activation, chronic inflammation, oxidative stress, and cellular and DNA damage, even in individuals with undetectable viral loads [1,2,3]. These disturbances could contribute to the development of comorbid conditions more prevalent among PLWH, such as cancer [4,5,6].

These phenomena may be explained by the persistent production of viral proteins that continue to be expressed despite viral suppression by ART [7,8]. Among these, the HIV-1 transactivator of transcription (Tat), which plays a central role in viral replication, also exhibits pathogenic effects as a multifunctional extracellular protein. Tat can exert immunomodulatory, genotoxic, and cytotoxic effects due to its ability to interact with various cellular receptors, including heparan sulfate proteoglycans, integrins, and CD26 [9,10,11]. This enables its binding to both infected and uninfected cells, leading to changes in inflammatory pathways, antioxidant gene expression, and the induction of DNA damage, as previously reported [12].

Although the Tat protein can be released and detected in serum, it is not fully immunogenic, and only a small proportion of individuals living with HIV develop an effective anti-Tat antibody response. Among them, those with detectable anti-Tat IgG antibodies have been reported to experience slower disease progression, suggesting a potential protective role against HIV-induced immunosuppression [13,14,15]. However, the specific effects of this humoral immune response, particularly in relation to oxidative stress-induced DNA damage and cytotoxicity, remain unclear.

In this context, we conducted a cross-sectional study in a cohort of Mexican PLWH receiving ART to explore the prevalence of anti-Tat IgG antibodies and their association with cytotoxic and genotoxic markers, including nuclear anomalies in buccal epithelial cells and oxidative DNA damage (8-OHdG), as well as immunological parameters such as CD4^+^ T cell counts. This study aims to contribute to understanding the complex immunopathogenic role of anti-Tat antibodies and their relevance in the post-ART era.

## 2. Results

To determine the prevalence of anti-Tat IgG antibodies, we developed an in-house ELISA. A biotin–streptavidin system was employed for signal amplification to enhance sensitivity. To reduce background noise, minimize matrix effects, and improve specificity, several critical factors were managed: EDTA serum sample treatment has been described as a strategy to inhibit complement-related interference [16,17], samples were clarified by ultracentrifugation, and a high dilution factor was used to reduce nonspecific binding. Background absorbance was defined using sera from healthy HIV-negative individuals, and a stringent cutoff value, the mean plus three standard deviations, was applied. Additionally, each assay plate included a semi-quantitative standard curve to estimate antibody levels in arbitrary ELISA units per milliliter (EU/mL).

**Figure 1:**
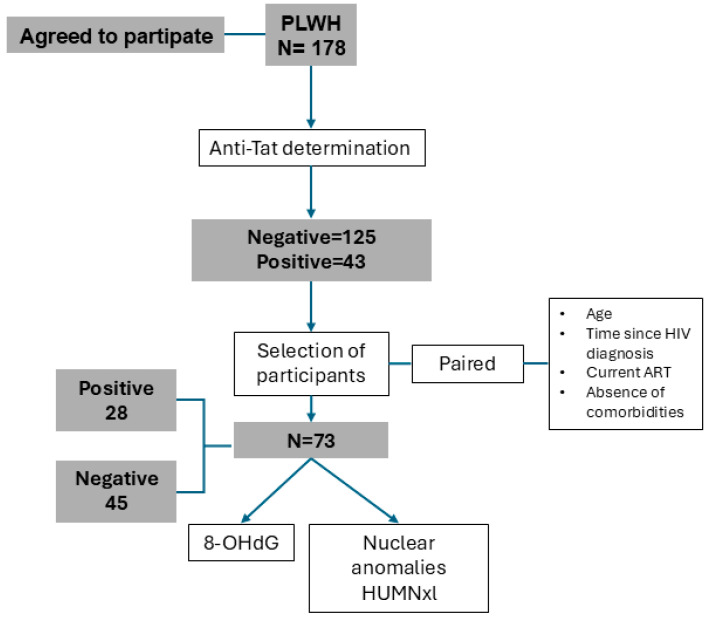
Algorithm for the selection of study participants.

Our whole sample studied to determine HIV anti-Tat IgG prevalence was 178 participants (Figure 1). Among them, 43 individuals (24.2%) were positive for anti-Tat antibodies. Notably, age showed a statistically significant difference between antibody-positive and negative individuals, with median ages of 33 and 39 years, respectively (*p* = 0.015). Although CD4+ T lymphocyte counts tended to be lower in the antibody-positive group, this difference was not statistically significant (*p* = 0.063). However, the proportion of individuals with ≥500 cells/µL was significantly lower in the positive group compared to the negative group (*p* = 0.023). CD4+ T cell count data were missing in 16 of 178 participants (8.98%) due to incomplete records. A full description of participant characteristics is provided in Table 1.

The median anti-Tat antibody level among seropositive individuals was 7.04 EU/mL, with an interquartile range of 33.64 EU/mL. Detailed data from HIV-positive participants are presented in Table 2. A noteworthy finding is that four individuals exhibited high anti-Tat antibody concentrations, exceeding the 90th percentile (above 628.95 EU/mL). These individuals had a significantly shorter time since HIV diagnosis (median of 1 year) compared to the other anti-Tat antibody-positive participants (median of 4 years, *p* = 0.03). Interestingly, individuals with higher antibody levels showed lower CD4+ T lymphocyte levels; however, this difference was not statistically significant (*p* = 0.099).

**Figure 2:**
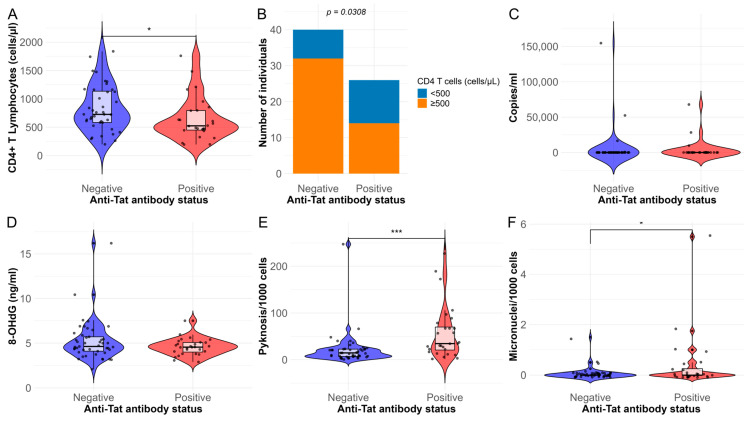
Comparison between anti-Tat positive and negative subgroups: (**A**) CD4+ T lymphocyte count, (**B**) proportion of individuals with CD4+ T lymphocyte counts ≥500 cells/µL, (**C**) viral load, (**D**) 8-hydroxy-2′-deoxyguanosine (8-OHdG) concentration, (**E**) pyknosis levels, and (**F**) micronuclei levels. *** = *p* < 0.001; * = *p* < 0.05.

In the selected subgroups (28 positive and 45 negative), the median age was 32 years in both groups (*p* = 0.654). The median duration of HIV infection was 4 years in both groups (*p* = 0.842). All the selected participants for both subgroups received ART consisting of bictegravir, tenofovir alafenamide, and emtricitabine. From all the variables analyzed, we observed that positive individuals have lower levels of CD4+ T lymphocytes than negatives (*p* = 0.045). Also, the proportion of individuals having ≥500 cells/µL was significantly lower (*p* = 0.031) in the anti-Tat positive group (Figure 2); CD4+ T cell count data were missing in 7 of 73 participants (9.6%) due to incomplete records. All the other characteristics were similar in both groups (Table 3).

**Figure 3:**
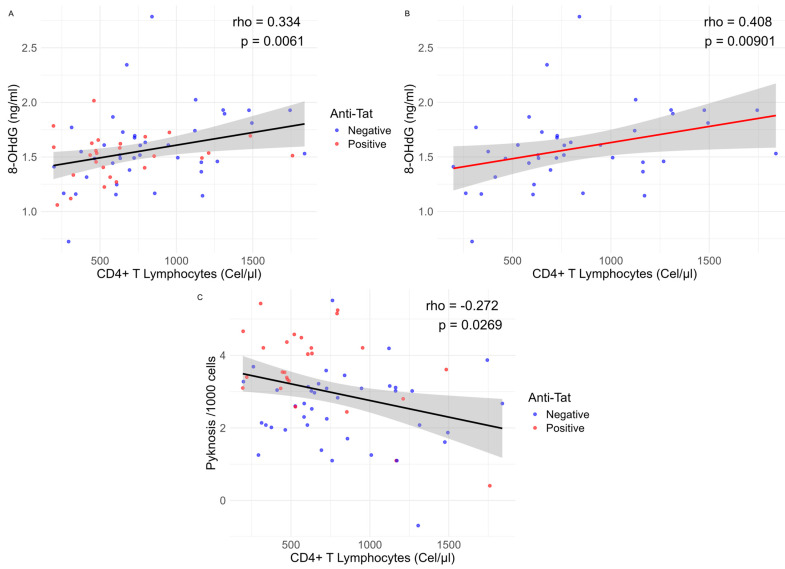
Spearman correlation plots of 8-OHdG (**A**) in all selected participants, (**B**) in anti-Tat antibody-negative participants only, and (**C**) pyknosis with CD4+ T lymphocytes in all selected participants. For visualization, values were transformed using the natural logarithm (log_e_).

Regarding the assessment of DNA damage tests. The 8-OHdG levels showed no statistically significant differences between groups, but there was a statistically significant positive correlation with CD4+ lymphocyte counts (rho = 0.334, *p* = 0.006). Subgroup analysis showed that this correlation was significant among anti-Tat-negative individuals (rho = 0.408, *p* = 0.009, Figure 3), while no correlation was found among anti-Tat-positive individuals (rho = 0.106, *p* = 0.6).

In the evaluation of nuclear anomalies, we observed that positive individuals showed higher levels of pyknosis (*p* = 0.0001511) than negative individuals (Figure 2), also there was a statistically significant negative correlation with CD4+ T lymphocyte counts (rho= −0.272, *p* = 0.027), suggesting that when pyknosis increases, there are a decrease in CD4+ T lymphocytes (Figure 3). In addition, we observed that positive individuals have a small, but statistically significant, increase in micronuclei levels (*p* = 0.026).

## 3. Discussion

To our knowledge, this is the first study that evaluates the prevalence of the anti-Tat immune response in Mexico. The prevalence of IgG anti-Tat antibodies in our population was 24.3%, like to the prevalence reported by other authors. For instance, Tripiciano et al. (2021) reported a prevalence of 19.0% in a cohort of 148 cART-treated individuals [18]. Similarly, Kashi and colleagues (2009) found a prevalence of 14% among PLWH in India. Nicoli and collaborators (2016) reported a slightly higher prevalence of 36% in a cohort of 96 cART-naïve individuals with chronic HIV infection from Tanzania [19]. Although there are some differences in prevalence across populations, all reported rates fall within a relatively narrow range. This supports the idea that the HIV Tat protein is not fully immunogenic, likely due to its unstable and multifunctional nature, as previously described [10].

Regarding antibody concentrations, we found that a small portion of anti-Tat-positive individuals had elevated antibody levels, and those individuals had a shorter time since HIV diagnosis. This finding is consistent with the study by Kashi et al., who reported that 7% of anti-Tat positive individuals showed higher antibody titers, principally IgG1 [20], which are elevated during early HIV infection [21]. These observations suggest that, in the early stages of HIV infection, higher anti-Tat titers may be associated with immune activation and B-cell stimulation [22]. However, anti-Tat antibody levels tend to decrease over time, as has been previously reported [13,23].

On the other hand, we observed that anti-Tat-positive individuals were younger than anti-Tat-negative individuals. This finding is consistent with a longitudinal cohort study involving 252 Italian individuals with the diagnosis of HIV infection, which reported a statistically significant difference in age between positive and negative anti-Tat individuals (25 vs. 29, respectively, *p* = 0.042) [14]. Krone et al. found that the frequency of anti-Tat antibodies was higher in children than in adults (55% vs. 36%), further suggesting age-related differences [23]. In contrast, other studies, such as those by Tripiciano et al., reported no significant differences between groups [18]. These results are not conclusive regarding the effect of age on the anti-Tat immune response, but they may suggest that younger individuals have more effective antigen recognition and antibody production. Nonetheless, these findings should be interpreted with caution, as the age differences observed are modest, and the biological impact may be limited.

We observed a tendency for anti-Tat-positive individuals to exhibit lower CD4+ T cell counts and a smaller proportion of participants with ≥500 cells/µL, which remained statistically significant in the subgroup analysis. This finding contrasts sharply with previous reports, such as those by Bellino et al. (2014), a cohort study of art-naïve individuals, and Tripiciano et al. (2021), a cohort study of individuals receiving ART. These studies did not report significant differences in CD4+ counts between anti-Tat-positive and anti-Tat-negative groups at baseline. However, they reported a protective effect against CD4+ T cell decline over time [15,18]. It is essential to highlight that this effect was observed in individuals compared to their baseline values, rather than through direct comparisons between anti-Tat-positive and anti-Tat-negative groups. These discrepancies may be attributed to differences in participant characteristics, the types of ART regimens used, and the study design, as the present study is cross-sectional rather than longitudinal and therefore cannot assess CD4+ trends over time.

Although the HIV Tat protein has been associated with oxidative stress and DNA damage, no previous studies have explored the potential impact of anti-Tat antibodies on HIV-related DNA damage. Our study did not show a significant relationship between anti-Tat antibody levels and the oxidative DNA damage marker 8-OHdG in selected antibody-positive and negative individuals. However, the observed correlation between 8-OHdG levels and CD4+ T cell counts suggests that anti-Tat antibodies might contribute indirectly to oxidative stress and DNA damage. Our finding is consistent with that reported by Shmakova et al. (2023), who reported a positive correlation between CD4+ T cell percentages and serum Tat protein levels in PLWH [24]. This effect may stem from the sustained expression of Tat by infected cells, which is released into the bloodstream and extracellular environment. Once extracellular, Tat may be internalized by bystander cells, impairing their antioxidant defenses and leading to increased oxidative stress and subsequent DNA damage [7,11]. The absence of correlation between 8-OHdG levels and CD4+ T cell counts in anti-Tat-positive individuals may reflect the protective role of anti-Tat antibodies, which could neutralize circulating Tat protein and prevent cell uptake. In contrast, anti-Tat-negative individuals may remain susceptible to Tat-induced oxidative stress due to ongoing exposure to extracellular Tat.

With respect to nuclear anomalies, a battery of DNA damage and cytotoxicity markers that may reflect bystander effects resulting from systemic exposure to diverse factors. Previous studies have reported that people with HIV exhibit higher levels of genomic instability, such as nuclear buds, binucleated cells, and micronuclei, than healthy individuals [2,25]. However, it has not been established whether those parameters differ with respect to anti-Tat humoral immune status. We found that anti-Tat antibody-positive individuals exhibited higher levels of pyknosis, which may suggest a role for anti-Tat antibodies in mediating antibody-dependent cellular cytotoxicity (ADCC), potentially contributing to the lower CD4+ T cell counts observed in these individuals. It has been reported that the immune response against Tat could promote ADCC, as reported by Kulkarni et al. (2017). In a study conducted in an Indian population of ART-naïve PLWH, they found that a proportion of sera from non-progressors recognized both Env and Tat epitopes. These sera also exhibited strong ADCC responses, as measured by a natural killer (NK) cell activation assay. Moreover, anti-Tat-mediated ADCC correlated with lower viral loads, although no significant differences in CD4+ T cell counts were observed [26]. More recently, Martino et al. (2024) reported that anti-Tat-positive sera from PLWH demonstrated high cytotoxic capacity, evaluated by a fluorometric assay [27].

ADCC is a mechanism mainly IgG-mediated, in which Fcγ receptors (FcγRs) recognize the Fc antibody region in effector cells such as natural killer (NK) cells. This interaction allows effector cells to bind to antigens expressed on the surface of target cells, resulting in the release of perforins and granzymes that induce cell death [28]. In this context, Tat is a multifunctional protein secreted by HIV infected T lymphocytes that could interact with cellular receptors such as heparan sulfate and integrins, which are present in many cellular types, and CD26 on T lymphocytes [9]. Consequently, the HIV Tat protein could be localized in infected and uninfected cells, potentially allowing antibodies to recognize and bind to Tat, facilitating NK cell engagement and triggering ADCC. This may explain the lower CD4+ counts and the higher cytotoxic damage observed in our study. Since ADCC is an immune mechanism that eliminates damaged or infected cells, this effect could reflect the efficient removal of presumed infected cells (either infected or antigen-bound), which may explain its protective role in HIV infection progression, as previously reported by different studies.

Nevertheless, given the absence of Tat protein quantification, we cannot exclude the possibility that some of the observed effects might be related to the Tat protein itself and its various effects previously reported. The presence of anti-Tat antibodies could potentially reflect prior exposure to higher levels of Tat, suggesting that positive individuals may have higher Tat levels compared to negative individuals, as is generally stated [29], but this relationship has not been studied. In relation to the role of Tat in apoptosis, some in vitro studies have suggested that extracellular Tat, released by infected cells, may interact with microtubules through tubulin binding and could activate mitochondria-dependent apoptotic pathways, possibly involving the pro-apoptotic Bcl-2 family protein Bim [30].

Regarding micronuclei, a small but statistically significant increase was observed in anti-Tat-positive individuals. This may reflect higher circulating Tat protein levels not fully neutralized by antibodies or an inflammatory response triggered by antibody-mediated mechanisms such as immune complex formation and complement activation [31,32]. These processes may contribute to chronic inflammation and subsequent DNA damage. In addition, Tat has been implicated in the production of oxidative stress through downregulation of antioxidant pathways and increased mitochondrial ROS production that has been related to DNA damage [11]. However, further studies are needed to clarify these mechanisms.

It is important to note that these results may reflect a transient effect of the immunological response against the virus, suggesting a mechanism that favors viral clearance through the elimination of infected cells, rather than indicating a deleterious effect of the presence of anti-Tat antibodies. But further studies are needed to evaluate the real long-term effects of anti-Tat antibodies and their potential protective effects in the context of ART-treated individuals.

This study has some limitations. Its cross-sectional design restricts the ability to infer causal relationships or temporal patterns in anti-Tat antibody responses and their associations with outcomes. Circulating Tat protein levels were not directly measured, and we did not assess the neutralizing capacity, avidity, or subclass distribution of anti-Tat antibodies, all of which could help understand their functional role. Furthermore, although our results suggest the potential involvement of antibody-dependent cellular cytotoxicity (ADCC) in CD4+ T cell loss and cytotoxic damage, ADCC activity was not directly measured, limiting mechanistic interpretation. These limitations highlight the need for further studies to explore the functional properties of anti-Tat antibodies, their relationship with Tat protein levels, and their potential contribution to immune modulation in people living with HIV.

## 4. Materials and Methods

In this cross-sectional study, 178 individuals living with HIV were recruited to determine the prevalence of anti-Tat IgG antibodies using an in-house ELISA. Participants were enrolled through the Infectiology Services at Hospital General Regional 110 and UMAE Hospital de Especialidades, from the Centro Médico Nacional de Occidente, both part of the Instituto Mexicano del Seguro Social (IMSS). Based on the results of anti-Tat serostatus, subgroups of 28 anti-Tat-positive and 45 anti-Tat-negative participants were selected. All participants were informed about the study’s purpose, potential risks, and benefits, and each patient signed a written consent form in accordance with the local and international regulations.

They answered a questionnaire regarding their habits, medical treatments, and comorbidities. Also, patients provided us with consent to examine their medical records as part of the study. The study was approved by the institutional Local Committee on Health Research (R-2023-1305-021).

Once anti-Tat serostatus was determined, we selected 25 anti-Tat-positive individuals who were free from opportunistic infections, AIDS-associated conditions, diabetes, rheumatic or inflammatory diseases, and acute or chronic infections. All were receiving ART consisting of bictegravir, tenofovir alafenamide, and emtricitabine. Participants started ART between one and three months after HIV diagnosis. A comparison group of 48 anti-Tat negative individuals was then selected, matched by age and time since HIV diagnosis, and meeting the same inclusion criteria. This approach was used to establish a homogeneous study population for the quantification and analysis of 8-OHdG levels and nuclear anomalies.

### 4.1. Sample Collection and Preparation

We collected serum and oral mucosa cell samples for all participants. Serum was collected using SST™ II Advance (BD Vacutainer Cat: 368965) tubes. After clotting, the samples were centrifuged at 3500 rpm for 10 min, and the serum was separated and stored at −70 °C. Additionally, samples from 12 HIV-negative individuals (with prior informed consent) were collected to serve as negative controls.

Oral mucosal samples were obtained after participants rinsed their mouths with water. Cells were collected from both the right and left cheeks using culture swabs and then resuspended in sterile water. The cells were transferred to 1.5 mL tubes and centrifuged at 4000 rpm for 10 min. The supernatant was discarded, and the cell pellet was resuspended in 150 µL of sterile water. The cells were then smeared onto microscope slides, air-dried, and fixed in absolute ethanol for 48 h. Once fixed, the cells were stained with acridine orange and stored for further analysis.

### 4.2. HIV Anti-Tat ELISA

An in-house ELISA was developed using 96-well plates coated with 100 ng of clade B Tat protein (Mybiosource, San Diego, CA USA, Cat: MBS538314)in 100 µL of BupH™ carbonate-bicarbonate buffer (Thermo Scientific™, Waltham, MA USA , Cat: 28382) per well and incubated overnight at 4 °C. Plates were blocked with SuperBlock™ buffer (Thermo Scientific™, USA, Cat:37515). Serum samples were pretreated with 10 µL of 0.2 M EDTA per 190 µL of sample (10 Mm), centrifuged (10,000 rpm × 10 min), and then diluted 1:200 in blocking buffer containing 0.5% (sample and standard diluent) Tween 20. A standard curve was generated using serial dilutions of polyclonal anti-Tat antibody (Abcam, Cambridge, UK, Cat: ab43014) in diluent with 1:200 pooled HIV negative serum. Samples and standards (100 µL) were added to the plate and incubated for 1 h, followed by five PBS-Tween washes. Then 100 µL of a 1:1 mix of cross-adsorbed biotinylated anti-human and anti-rabbit IgG secondary antibodies (1:120,000) were added (Invitrogen, Carlsbad, CA USA, Cat: A18815 and Cat: 65-6140) incubated for 1 h at room temperature with gentile shaking, then washed five times (3 min each), followed by the addition of Pierce™ High Sensitivity Streptavidin-HRP (Thermo Scientific, Cat: 21130) (1:100,000), with 1 h incubation followed by five washes (3 min each). Color development was initiated by adding 100 µL of 1-Step Turbo TMB (Thermo Scientific, Cat: 34024) and incubated protected from light for 30 min. The reaction was stopped by adding 100 µL of stop solution (Thermo Scientific, Cat: N600), and absorbance was read at 450 nm using a Multiskan™ GO Microplate Photometer (Thermo Scientific, Cat: 51119200). The positivity threshold was set as the mean OD of 12 HIV-negative controls plus three standard deviations. Concentrations were calculated using a four-parameter standard curve with SkanIt 6.1 software for microplate readers (Thermo Scientific) and expressed as arbitrary ELISA units. Positive, negative control and standard curve were performed in each 96-well plate.

### 4.3. 8-OHdG Determination

For 8-Hydroxy-2′-deoxyguanosine measurement, the 8-hydroxy 2-deoxyguanosine ELISA Kit (Abcam, Cambridge, UK, Cat: ab201734) was employed. Serum samples were diluted 1:20 (*v*/*v*) with the Sample and Standard Diluent provided in the kit. A standard curve was performed using serial dilutions of the 8-OHdG standard according to the kit protocol. The 50 µL of either standard or diluted sample was added to each well of the 96-well microplate. Subsequently, 50 µL of diluted HRP-conjugated anti-8-OHdG monoclonal antibody was added to each well. Plates were incubated for 1 h at room temperature and then washed 4 times. Then 100 µL of TMB was added to each well and allowed to develop for 30 min. Finally, 100 µL of stop solution was added. Plates were read at 450 nm using Multiskan™ GO Microplate Photometer (Thermo Scientific, Cat: 51119200), and unknown samples were interpolated from the standard curve.

### 4.4. Nuclear Anomalies Quantitation

Mucosal stained samples were observed with fluorescence microscopy using an Olympus BX51 microscope (Olympus Corporation, Tokyo, Japan) at 100× magnification. For each sample, 2000 cells were analyzed to evaluate the presence of nuclear anomalies (micronuclei, binucleated cells, nuclear buds, karyorrhexis, karyolysis, and pyknosis). Scoring was performed according to the Human Micronucleus Cytome (HUMNxl) scoring criteria.

### 4.5. Statistical Analysis

Quantitative variables are presented as medians and interquartile ranges. Categorical variables are described using frequencies and percentages. Pairwise comparisons were conducted using the Mann–Whitney U test for quantitative data, and the Chi-squared test or Fisher’s exact test for categorical data, as appropriate. Correlations were assessed using Spearman’s rank correlation coefficient. A *p*-value of <0.05 was considered statistically significant. Statistical analyses were performed using RStudio (version 2024.12.1 Build 563; Posit, PBC, Boston, MA, USA) with R version 4.3.2 (2023-10-31, ucrt).

## 5. Conclusions

This study, the first in Mexico to assess anti-Tat IgG antibody prevalence, found that 24.3% of people living with HIV (PLWH) were anti-Tat positive. Contrary to initial expectations, these ART-treated individuals exhibited higher levels of pyknosis and lower CD4+ T cell counts, rather than reduced DNA damage. One possible explanation could be the involvement of ADCC. This effect could reflect enhanced viral clearance and contribute to immune modulation, suggesting a more complex role for anti-Tat antibodies in HIV pathogenesis. Further studies are needed to clarify this dual potential.

## Figures and Tables

**Table 1:** Demographic and clinical characteristics of participants.

Variable		Anti-Tat Antibody Status		
		Negative (135)	Positive (43)	*p*-Value
Sex	Female	16 (9.0%)	7 (3.9%)	0.309
	Male	119 (66.9%)	36 (20.2%)	
Age (years)		39.0 (19.0)	33.0 (17.0)	0.015
Time since diagnosis (years)		5.0 (10.0)	4.0 (8.0)	0.519
ART	Yes	128 (71.9%)	43 (24.2%)	0.198
	No	7 (3.9%)	0 (0.0%)	
Viral load	Undetectable	121 (68.0%)	35 (4.5%)	0.184
	Detectable	14 (7.9%)	8 (4.5%)	
	Copies/mL *	103,526.0 (501,549.0)	44,182.0 (108,148.0)	0.57
CD4+ T lymphocytes	Cells/µL	755.0 (526.0)	607.0 (435.0)	0.063
	≥500 cells/µL	96 (59.3%)	22 (13.6%)	0.023
	0–499 cells/µL	28 (17.3%)	16 (9.9%)	

Data are presented as medians (interquartile ranges). Comparisons between groups were performed using the Mann–Whitney U test. Viral load values (copies/mL) are reported only for individuals with detectable levels *.

**Table 2:** Demographic and clinical characteristics of anti-Tat antibody-positive individuals according to antibody concentration levels.

Variable		Anti-Tat Antibody Concentration		
		Lower 39 (90.7%)	Higher 4 (9.3%)	*p*-Value
Sex	Female	7.0 (16.3%)	0 (0.0%)	0.999
	Male	32.0 (74.4%)	4 (9.3%)	
Age (years)		37.5 (20.0)	33.5 (24.0)	0.606
Time since diagnosis (years)		5.0 (10.0)	1.0 (4.0)	0.03
ART	Yes	39.0 (9.7%)	4 (9.3%)	0.999
	No	0 (0.0%)	0 (0.0%)	
Viral load	Undetectable	32.0 (74.4%)	3 (7.0%)	0.488
	Detectable	7.0 (16.3%)	1 (2.3%)	
	Copies/mL *	52,389.0 (336,203.0)	67,861.0 (N/A)	0.999
CD4+ T lymphocytes (cells/µL)		739.0 (567.0)	480.5 (83.0)	0.099

Data are presented as medians (interquartile ranges) or frequencies (percentages). Comparisons between groups were performed using the Mann–Whitney U test. Viral load values (copies/mL) are reported only for individuals with detectable levels *.

**Table 3:** Characteristics of selected individuals regarding anti-Tat antibody status.

Variable			Anti-Tat Antibody Status		
			Negative (45)	Positive (28)	*p*-Value
Sex		Female	3 (4.1%)	3 (4.1%)	0.669
		Male	42 (57.5%)	25 (34.2%)	
Age			32.0 (20.0)	32.0 (14.0)	0.654
Time since diagnosis (years)			4.0 (6.0)	4.0 (4.0)	0.842
Total cholesterol (mg/dL)			179.0 (48.0)	170.0 (38.0)	0.093
HDL (mg/dL)			39.5 (15.0)	40.0 (11.0)	0.966
LDL (mg/dL)			100.0 (38.0)	96.0 (31.0)	0.204
Triglycerides (mg/dL)			139.0 (87.0)	130.0 (118.0)	0.6
Viral load	Undetectable		40 (54.8%)	23 (31.5%)	0.492
	Detectable		5 (6.8%)	5 (6.8%)	
	Copies/mL *		16,545.0 (103,162.0)	9761.0 (47,875.0)	0.841
CD4+ T lymphocytes	Cells/µL		726 (571)	524 (367)	0.045
	≥500 cells/µL		32 (48.5%)	14 (21.2%)	0.031
	0–499 cells/µL		8 (12.1%)	12 (18.2%)	
8-OHdG (ng/mL)			4.6 (1.75)	4.5 (1.21)	0.326
Micronuclei			0.0 (0.0)	0.0 (0.25)	0.026
Nuclear buds			1.0 (1.50)	1.5 (2.25)	0.211
Binucleated cells			0.0 (0.0)	0.0 (0.0)	0.205
Pyknosis			14.5 (16.0)	34.37 (57.8)	0.0001
Condensed chromatin			76.5 (105.0)	97.0 (143.3)	0.343
Karyorrhexis			62.0 (197.5)	58.1 (69.6)	0.204
Karyolysis			2.25 (3.0)	0.5 (1.31)	0.067

Data are presented as medians (interquartile ranges) or frequencies (percentages). Nuclear anomalies are presented per 1000 cells. Comparisons between groups were performed using the Mann–Whitney U test. Viral load values (copies/mL) are reported only for individuals with detectable levels *.

## Data Availability

Raw data are available from the corresponding author upon request.
